# Exploratory Analysis of Quantitative CT Metrics for Predicting Tumor Aggressiveness and Nodal Metastasis in Head and Neck Squamous Cell Carcinoma: A Retrospective Cohort Study

**DOI:** 10.3390/cancers18111706

**Published:** 2026-05-23

**Authors:** Ingrid-Denisa Barcan, Dan Costachescu, Ademir Horia Stana, Alexandru Catalin Motofelea, Alexandra Christa Sima, Dana Emilia Movila, Nadica Motofelea, Tudor Ciocarlie, Eugen Radu Boia, Delia Ioana Horhat

**Affiliations:** 1 Department of Doctoral Studies, “Victor Babes” University of Medicine and Pharmacy Timisoara, Eftimie Murgu Square No. 2, 300041 Timisoara, Romania; ingrid.barcan@umft.ro (I.-D.B.); alexandru.motofelea@umft.ro (A.C.M.); 2 ENT Department, “Victor Babes” University of Medicine and Pharmacy Timisoara, Eftimie Murgu Square No. 2, 300041 Timisoara, Romania; eugen.boia@umft.ro (E.R.B.); horhat.ioana@umft.ro (D.I.H.); 3 ENT Department, Emergency City Hospital, 300254 Timisoara, Romania; 4 Department of Radiology and Medical Imaging, “Victor Babes” University of Medicine and Pharmacy Timisoara, Eftimie Murgu Square No. 2, 300041 Timisoara, Romania; 5 Department Medicine, Discipline of Radiology, Vasile Goldiş Western University, Liviu Rebreanu Boulevard, Nr. 86, 310414 Arad, Romania; 6 Centre for Molecular Research in Nephrology and Vascular Disease/MOL-NEPHRO-VASC, “Victor Babes” University of Medicine and Pharmacy Timisoara, Eftimie Murgu Square No. 2, 300041 Timisoara, Romania; sima.alexandra@umft.ro; 7 Department of Second Internal Medicine-Diabetes, Nutrition, Metabolic Diseases, and Systemic Rheumatology, “Victor Babes” University of Medicine and Pharmacy Timisoara, Eftimie Murgu Square No. 2, 300041 Timisoara, Romania; 8 Department VI-Cardiology, “Victor Babes” University of Medicine and Pharmacy Timisoara, Eftimie Murgu Square No. 2, 300041 Timisoara, Romania; dana.movila@umft.ro; 9 Department of Obstetrics and Gynecology, “Victor Babes” University of Medicine and Pharmacy Timisoara, Eftimie Murgu Square No. 2, 300041 Timisoara, Romania; nadica.motofelea@umft.ro; 10 Department VII Internal Medicine II, Discipline of Cardiology, “Victor Babes” University of Medicine and Pharmacy Timisoara, Eftimie Murgu Square No. 2, 300041 Timisoara, Romania; ciocarlie.tudor@umft.ro

**Keywords:** head and neck squamous cell carcinoma, computed tomography, tumor volume, histopathological grade, lymph node metastasis, tumor enhancement, Hounsfield units

## Abstract

Background: Preoperative assessment of Head and Neck Squamous Cell Carcinoma (HNSCC) aggressiveness is often hindered by the sampling errors of incisional biopsies. While Contrast-Enhanced Computed Tomography (CECT) is the standard for staging, its potential to serve as a non-invasive complementary radiological tool of the entire tumor volume remains underutilized. Objective: To evaluate the predictive performance of preoperative CECT-derived tumor volume, densitometric values, and morphological features as predictors of histopathological grade and lymph node metastasis (pN) in HNSCC. The primary outcome was predicting lymph node metastasis (pN+), and the secondary outcome was predicting histopathological grade. Methods: This retrospective observational study analyzed 42 patients with SCC of the oral cavity, larynx, or maxilla. Quantitative (3D volume, Hounsfield Units [HU], HU Delta) and qualitative (margins, lobulations, necrosis) CT parameters were correlated with definitive histopathology. Diagnostic performance was assessed using Receiver Operating Characteristic (ROC) curve analysis and Spearman’s rank correlation. Results: High-grade tumors (G2/G3) demonstrated significantly larger median volumes (18.1 vs. 2.9 cm^3^, *p* = 0.006), lower contrast density (55 vs. 68 HU, *p* = 0.010), and reduced vascular wash-in (23 vs. 30 HU Delta, *p* = 0.008) compared to G1 lesions. ROC analysis identified a volume threshold of ≥9.43 cm^3^ for high-grade disease (AUC = 0.865; sensitivity 67.6%, specificity 100%). For regional metastasis (pN+), tumor volume was the only significant predictor (25.4 vs. 6.2 cm^3^, *p* = 0.036), with an optimal cut-off of ≥6.76 cm^3^ (AUC = 0.769; sensitivity 100%). Strong negative correlations were observed between contrast enhancement and internal necrosis (r = −0.812, *p* < 0.001). Conclusions: Preoperative CECT parameters show promise as non-invasive imaging surrogates of HNSCC aggressiveness. A paradoxical reduction in contrast enhancement characterizes high-grade biology, reflecting disorganized neo-angiogenesis and internal hypoxia. Integrating 3D volumetric analysis and morphological markers shows potential as a complementary exploratory tool that, pending future prospective validation, may support risk stratification and surgical planning alongside traditional histopathological assessment.

## Simple Summary

Head and neck cancers are aggressive tumors where accurate preoperative assessment is critical for planning surgery and avoiding unnecessary overtreatment. Currently, surgeons rely on tissue biopsies to determine how aggressive a tumor is, but a small biopsy sample may not reflect the true nature of the entire tumor. In this study, we investigated whether routine preoperative CT scans already performed as standard of care could provide this information non-invasively. By analyzing the three-dimensional volume and the way tumors absorb contrast dye on CT imaging, we found that larger, more aggressive tumors paradoxically absorb less contrast, reflecting their disorganized internal blood supply and areas of oxygen deprivation. We identified specific volumetric thresholds that are correlated with both tumor aggressiveness and the spread to lymph nodes in the neck. These findings suggest that standard CT scans can serve as a complementary radiological tool, improving surgical planning without additional procedures.

## 1. Introduction

Head and neck cancer remains a significant global health burden, with recent epidemiological data estimating over 940,000 new cases and 480,000 deaths annually [1]. Head and Neck Squamous Cell Carcinoma (HNSCC) constitutes the vast majority of these malignancies, and its prognosis is heavily dependent on accurate staging and histological grading. Among the various prognostic factors, the presence of cervical lymph node metastasis is the most significant predictor of survival, often necessitating aggressive management such as elective neck dissection even in clinically node-negative necks [2,3]. Consequently, the preoperative assessment of tumor aggressiveness—defined by histological differentiation and metastatic potential—is critical for tailoring therapeutic strategies and minimizing overtreatment [4].

Currently, the gold standard for determining tumor grade and aggressiveness is the preoperative incisional biopsy [5]. While fundamental to diagnosis, this method has inherent limitations. HNSCC is a heterogeneous malignancy, and a small tissue sample taken from the superficial aspect of the tumor may not represent the biology of the deeper, invasive front, which is often the most prognostically relevant region [6]. A retrospective cohort analysis of oral cavity biopsies revealed that up to 60% of discordant diagnoses between the initial biopsy and the final resection specimen were attributable to sampling error [7]. Additionally, conventional histopathological grading systems are characterized by significant subjectivity, often demonstrating poor inter-observer reproducibility among pathologists [7]. These limitations highlight a potential clinical benefit for non-invasive methods that can characterize the biology of the entire tumor volume prior to surgical intervention.

Contrast-Enhanced Computed Tomography (CECT) is the workhorse of preoperative staging for HNSCC, primarily utilized to assess tumor dimensions and bony invasion according to TNM criteria [8]. However, CECT data contains physiological information that extends beyond morphology. The enhancement of tumor tissue after contrast administration is a direct reflection of neo-angiogenesis, a prerequisite for tumor growth and metastasis [9]. Early research established that contrast enhancement density is linearly related to tissue iodine concentration, acting as a surrogate marker for vascular physiology [10].

Recent investigations suggest that quantitative variations in CT imaging can offer insights into tumor biology, as aggressive, poorly differentiated tumors often exhibit disorganized vascularity and regions of micro-necrosis and hypoxia [11]. Advanced quantitative studies utilizing Dual-Energy CT (DECT), perfusion imaging, and radiomic texture analysis have successfully demonstrated correlations between altered perfusion parameters, hypoxia (such as HIF-1α expression), and tumor aggressiveness [12,13]. However, these advanced imaging techniques require specialized software and protocols that are not universally accessible. Consequently, there remains a significant gap in the literature regarding whether standard, widely available CECT parameters can provide comparable prognostic value. Evaluating these accessible metrics is essential for translating imaging-based precision oncology into routine clinical workflows. Therefore, imaging is positioned not to replace the gold standard of histopathology, but rather to serve as an additive, complementary tool.

Despite these advances, the use of standard preoperative CECT density values (Hounsfield Units) and specific morphological markers (such as volumetric analysis, lobulation and spiculation) to predict histological grade remains underutilized in routine clinical practice. Therefore, this study aims to evaluate the diagnostic accuracy of preoperative CT density, volumetric analysis, and morphological features as non-invasive predictors of histopathological grade and lymph node metastasis in patients with HNSCC. The primary endpoint was the prediction of regional lymph node metastasis (pN+), while the secondary endpoint was the association with histopathological grade.

## 2. Materials and Methods

### 2.1. Study Design and Setting

This study was designed as a retrospective, single-center observational analysis conducted at the ENT Department, Emergency City Hospital. The primary objective was to investigate the efficacy of preoperative contrast-enhanced computed tomography (CECT) parameters as non-invasive surrogates for histopathological grading and invasive front characteristics in patients with HNSCC. This research was reported in strict accordance with the Strengthening the Reporting of Observational Studies in Epidemiology (STROBE) statement to ensure methodological transparency and scientific rigor [14].

### 2.2. Patient Selection and Eligibility Criteria

A retrospective observational study was conducted using electronic and paper medical records from the Department of Otorhinolaryngology (ORL) and the Department of Oral and Maxillofacial Surgery at Spitalul Clinic Municipal de Urgență Timișoara. The institutional database was systematically reviewed to identify patients diagnosed with squamous cell carcinoma of the oral cavity, larynx, or maxilla between 2018 and 2022, representing a retrospective period of approximately 5 years. The inclusion criteria included: (1) Histopathologically confirmed primary squamous cell carcinoma of the oral cavity, larynx, or maxilla; (2) Availability of standard preoperative contrast-enhanced CT (native and venous phases) performed within 4 weeks prior to surgery; (3) Patients underwent primary surgical resection with neck dissection (allowing for definitive pN staging). The exclusion criteria included: (1) Previous radiotherapy or chemotherapy for head and neck cancer; (2) Severe CT artifacts (e.g., dental amalgam) obscuring the tumor; (3) Recurrent tumors; (4) Lack of definitive histological grading or nodal staging.

An initial pool of 115 patients was screened. Following the application of these criteria, 73 patients were excluded (primarily due to non-surgical management, missing standard CT protocols, or severe dental artifacts). Consequently, a final sample size of 42 eligible patients was enrolled in the study.

### 2.3. Radiological Acquisition and Quantitative Analysis

All imaging was performed using a multi-detector CT system (64-slice or greater) utilizing a standardized head and neck protocol. This included a native phase followed by a portal venous phase acquired 60–70 s after the administration of non-ionic iodinated contrast media. Quantitative image analysis was performed on a dedicated workstation by two senior radiologists, who remained blinded to the final histopathological results. Discrepancies in quantitative measurements or morphological scoring between the two radiologists were resolved through mutual consensus; if consensus could not be reached, a third senior radiologist was consulted. Volumetric assessment was conducted using the ellipsoid formula:

$$V\;=\;\frac{AP\;\times T\;\times CC\;\times \pi }{6}$$

where AP, T, and CC represent the maximum dimensions in the anteroposterior, transverse, and craniocaudal planes, respectively. Densitometric evaluation involved the manual placement of regions of interest (ROIs) within the solid, most enhancing portions of the tumor, deliberately avoiding areas of gross calcification or large adjacent vessels. While true 3D volumetric segmentation is highly accurate, the ellipsoid formula was deliberately chosen for its practicality, speed, and ease of integration into routine clinical workflows without the need for specialized software. The ellipsoid formula is an estimation and is not equivalent to true 3D slice-by-slice volumetric segmentation, particularly for highly irregular tumors. The mean Hounsfield Units (HU) were recorded for both phases, and the “HU Delta” (contrast wash-in) was calculated as:

*HU_Contrast_* − *HU_Native_*.

Qualitative Morphological Assessment.

Beyond quantitative metrics, a detailed morphological profile was established for each lesion. This included the assessment of the internal architecture (scored by the presence and extent of central necrosis) and the tumor boundary characteristics. Margins were classified as well-defined or ill-defined, and the presence of lobulations (convex, bulging contours) and spicules (fine, radiating linear opacities) was documented. These features were integrated into a composite margin score to quantify the radiological suspicion of an infiltrative growth pattern.

### 2.4. Histopathological Reference Standard

The surgical specimens served as the definitive reference standard. Following standardized fixation and sectioning, a specialized pathologist—blinded to the radiological findings—graded the tumors as G1 (well-differentiated), G2 (moderately differentiated), or G3 (poorly differentiated) based on the WHO classification. A meticulous examination of the invasive front was conducted to identify the presence of Lymphatic (L), Vascular (V), and Perineural (Pn) invasion. Pathological nodal status (pN) was determined through the examination of cervical lymphadenectomy specimens, categorizing patients into pN0 (node-negative) and pN+ (node-positive) groups. Histopathological grading was performed by a specialized head and neck pathologist. To mitigate observer bias, difficult or borderline cases were reviewed by a second pathologist, and a final grade was established by consensus.

### 2.5. Ethical Considerations

The study protocol was conducted in full compliance with the ethical principles of the Declaration of Helsinki [15]. Formal approval was obtained from the Research Ethics Committee of the “Victor Babeș” University of Medicine and Pharmacy Timișoara (Approval No. 18/23.02.2026). Institutional authorization for access to patient records was granted by Spitalul Clinic Municipal de Urgență Timișoara (Authorization No. E-1316/20.03.2026). Given the retrospective and observational nature of the study, individual informed consent was waived; all patient data were fully anonymized prior to analysis.

### 2.6. Statistical Analysis

Data analysis was performed using SPSS version 26.0 (IBM Corp., Armonk, NY, USA). The statistical plan was designed to evaluate the primary endpoint (prediction of lymph node metastasis, pN+) and the secondary/exploratory endpoint (association with histopathological grade). The normality of continuous variables was assessed using the Shapiro–Wilk test. As the data demonstrated a non-parametric distribution, continuous variables are reported as medians and interquartile ranges (IQR). Comparisons between histological grades and nodal status were performed using the Mann–Whitney U test for continuous variables and the Chi-square or Fisher’s exact test for categorical variables. The correlation between CT features and histopathological factors was evaluated using Spearman’s rank correlation coefficient (r).

To evaluate the diagnostic performance of tumor volume and density, Receiver Operating Characteristic (ROC) curve analysis was employed, with the Area Under the Curve (AUC) reported alongside 95% Confidence Intervals (CIs). Optimal diagnostic thresholds were identified via the Youden Index [16]. Sensitivity and specificity are reported with 95% CIs calculated using the exact binomial (Clopper–Pearson) method. Due to the limited number of events in this exploratory cohort (only 9 pN+ cases), multivariate logistic regression was not performed to avoid severe statistical overfitting (violating the standard requirement of at least 10 events per predictor variable). Furthermore, given the hypothesis-generating nature of this study, *p*-values were not adjusted for multiple comparisons. A two-tailed *p*-value of <0.05 was considered statistically significant.

## 3. Results

The baseline demographic, clinical, and radiographic characteristics of the 42 study participants are summarized in Table 1. The cohort was predominantly male (83.3%) with a median age of 61 years (IQR: 54–67). The oral cavity was the most frequent anatomical site, representing 76.2% of cases. Histopathological analysis revealed that the vast majority of tumors were moderately differentiated (G2, 85.7%), while the pathological T-stage was relatively evenly distributed between early (T1/T2, 54.5%) and advanced (T3/T4, 45.5%) disease. Radiographically, the median preoperative tumor volume was 16.1 cm^3^, and morphological assessment highlighted a high prevalence of lobulations (81%) and ill-defined margins (78.6%) across the cohort.

### 3.1. Preoperative CT Features as Predictors of Histological Grade

The associations between preoperative CT characteristics and histopathological grade, categorized as low-grade (G1) versus high-grade (G2/G3), are detailed in Table 2. High-grade tumors exhibited a significantly larger median volume compared to their low-grade counterparts (18.1 cm^3^ vs. 2.9 cm^3^, *p* = 0.006). Furthermore, high-grade lesions demonstrated significantly lower contrast density (median 55 HU vs. 68 HU, *p* = 0.010) and lower vascular wash-in (HU Delta median 23 vs. 30, *p* = 0.008). Morphologically, high-grade tumors were significantly more likely to present with higher margin scores (*p* = 0.004) and lobulated contours (*p* = 0.040). Ill-defined margins and spiculated borders were observed in 83.8% and 51.4% of high-grade tumors, respectively, though these differences did not reach statistical significance (*p* > 0.05).

**Figure 1:**
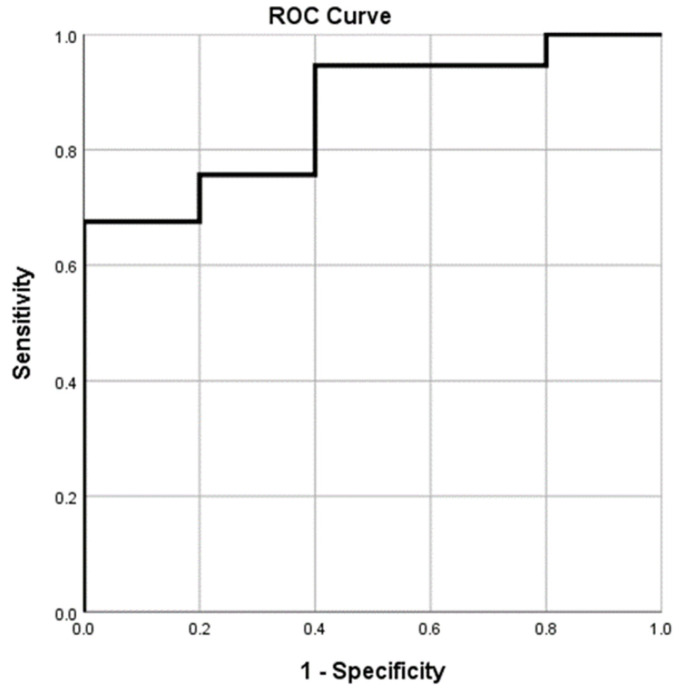
Receiver Operating Characteristic (ROC) curve analysis of preoperative tumor volume for the prediction of high histopathological grade (G2/G3) in HNSCC.

ROC curve analysis was conducted to evaluate the diagnostic performance of preoperative tumor volume in predicting high histopathological grade (G2/G3). Tumor volume demonstrated strong discriminative ability, yielding an AUC of 0.865 (95% CI: 0.73–1.00, *p* = 0.009). Utilizing Youden’s index, the optimal volume cut-off threshold for identifying high-grade tumors was determined to be ≥9.43 cm^3^. At this volumetric threshold, the model achieved a sensitivity of 67.6% (95% CI: 50.2–82.0%) and a specificity of 100% (95% CI: 54.9–100%), as in Figure 1.

### 3.2. Association of CT Features with Lymph Node Metastasis (pN)

The relationship between preoperative CT characteristics and regional lymph node metastasis is presented in Table 3. Among the evaluated parameters, preoperative tumor volume was the only metric significantly associated with nodal status; patients with histopathologically positive lymph nodes (pN+) had a significantly larger median tumor volume compared to those without nodal involvement (pN0) (25.4 cm^3^ vs. 6.2 cm^3^, *p* = 0.036). Conversely, CT density values (both native and contrast) and qualitative morphological features, including margin type and necrosis, did not demonstrate statistically significant differences between the node-negative and node-positive groups.

**Figure 2:**
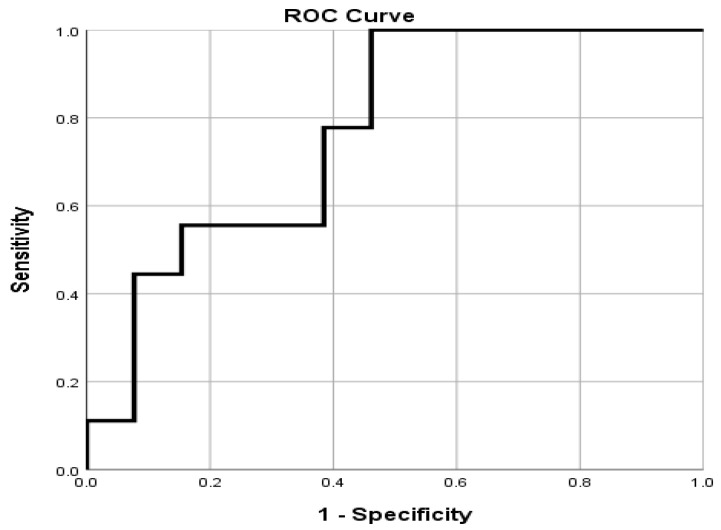
ROC curve for tumor volume as a predictor of lymph node metastasis.

A subsequent ROC curve analysis evaluated the capacity of preoperative tumor volume to predict regional lymph node metastasis (pN+). Tumor volume proved to be a significant predictor, yielding an AUC of 0.769 (95% CI: 0.571–0.968, *p* = 0.035). Application of Youden’s index identified an optimal cut-off threshold of ≥6.76 cm^3^. At this volume, the model demonstrated a sensitivity of 100% (95% CI: 66.4–100%) and a specificity of 53.8% (95% CI: 25.1–80.8%), as in Figure 2. It is important to note that the specificity for this threshold was modest (53.8%). Given the wide confidence intervals and the small number of positive events (*n* = 9), this high sensitivity must be interpreted strictly as an exploratory finding rather than a clinically reliable metric.

### 3.3. Correlation Between Preoperative CT Features and Internal Architecture

Spearman’s rank analysis revealed significant relationships between tumor volume, CT density, and morphological features (Table 4). Tumor volume demonstrated a strong positive correlation with necrosis score (r = 0.626, *p* < 0.001) and margin score (r = 0.594, *p* < 0.001), while showing a significant negative correlation with mean contrast density (r = −0.554, *p* < 0.001). Among morphological indicators, worse margin scores strongly correlated with higher necrosis scores (r = 0.724, *p* < 0.001). Furthermore, both native and contrast densities exhibited strong negative correlations with the extent of necrosis (r = −0.808 and r = −0.812, respectively; *p* < 0.001). Finally, contrast wash-in (HU Delta) was negatively correlated with necrosis score (r = −0.583, *p* < 0.001) and margin score (r = −0.494, *p* = 0.001).

## 4. Discussion

The accurate preoperative characterization of tumor aggressiveness in HNSCC remains a pivotal challenge in optimizing surgical planning. This exploratory study suggests that routine Contrast-Enhanced Computed Tomography (CECT) parameters may serve as promising, non-invasive imaging surrogates for histopathological grading and metastatic potential. Our findings indicate that quantitative volumetric analysis and contrast wash-in dynamics are associated with tumor differentiation and regional lymph node status. Specifically, we observed that larger, biologically aggressive tumors exhibited a paradoxical reduction in contrast enhancement potentially indicative of internal necrosis and hypoxia alongside infiltrative morphological patterns. These data hypothesize that standard CECT could offer valuable prognostic information to complement, rather than replace, traditional biopsy and TNM staging.

A salient finding of this study is the superiority of three-dimensional (3D) tumor volume over conventional linear dimensions in predicting cervical lymph node metastasis. While the current TNM staging system relies heavily on tumor diameter (T-stage) and depth of invasion (DOI), our data indicates that total tumor volume provides a more accurate representation of the total clonogenic burden. This aligns with the concept described in several studies, where a larger tumor volume serves as a surrogate for a higher number of clonogenic cells, increasing the biological probability of metastasis and treatment resistance [17,18,19,20].

In our cohort, a tumor volume exceeding 6.76 cm^3^ was highly predictive of lymph node metastasis. This threshold sits logically between the values reported for early and advanced diseases in the literature. For instance, Yang et al. demonstrated that a metabolic tumor volume of ≥4.3 cm^3^ was a significant predictor of occult cervical metastasis in early-stage (cT1-T2) tongue squamous cell carcinoma [21]. Conversely, studies focusing on advanced stages, such as the 20 cm^3^ cut-off reported by Joo et al. [22] and the 23 mL cut-off by Chen et al. [23] for T4a carcinomas, propose much higher thresholds. The fact that our optimal cut-off of 6.76 cm^3^ lies slightly higher than Yang et al.’s early-stage threshold, but significantly lower than the thresholds for advanced T4 tumors, accurately reflects the staging diversity (T1–T4) and mixed anatomical distribution of our study population. Nevertheless, the consensus across these studies confirms that 3D volume is a robust prognosticator that captures the biological burden more effectively than T-stage alone.

When evaluating our 6.76 cm^3^ threshold for nodal metastasis, it yielded a sensitivity of 100% [95% CI: 66.4–100%] and a specificity of 53.8% [95% CI: 25.1–80.8%]. It is crucial to interpret this 100% sensitivity with extreme caution, as it is highly likely overestimated due to the small number of outcome events (9 pN+ cases) in our cohort. However, from a clinical perspective, an imaging test with high sensitivity and moderate specificity could still hold value as a “rule-out” tool. In HNSCC, the clinical priority is to avoid missing occult metastases (false negatives). A false positive merely results in an elective neck dissection—which is often the standard of care—whereas a false negative leads to undertreatment and regional recurrence. Furthermore, while our study highlights tumor volume, other studies emphasize Depth of Invasion (DOI) or tumor thickness as the paramount predictors of nodal disease. Because our sample size precluded multivariate analysis, we cannot definitively conclude that 3D volume outperforms these traditional histopathological metrics; rather, it should be viewed as an additive preoperative indicator.

Perhaps the most distinct finding of our study is the inverse relationship between contrast enhancement and histological grade. High-grade tumors (G2/G3) exhibited significantly lower mean contrast density and reduced wash-in (HU Delta) compared to well-differentiated lesions. While intuitively one might expect aggressive tumors to be hypervascular, our findings reflect the complex physiology of tumor neo-angiogenesis. As described by Folkman et al., rapidly proliferating tumors undergo an “angiogenic switch,” recruiting new vessels that are structurally defective, convoluted, and blind-ended [24]. These immature vessels are highly permeable, leading to fluid leakage into the interstitial space [10]. This leakage increases the interstitial fluid pressure (IFP) within the tumor core, which can mechanically compress vessels and impede the delivery of contrast agents during the venous phase. Furthermore, aggressive tumors often outgrow their blood supply, leading to regions of chronic hypoxia and micro-necrosis that appear hypodense on CT. This radiological phenotype is supported by Bogowicz et al., who found that specific CT radiomic texture features correlated directly with molecular markers of hypoxia (Osteopontin) and angiogenesis (VEGF) [25]. Thus, “low density” on preoperative CT should not be interpreted as a lack of vascularity, but rather as a radiological marker of disorganized, hypoxic, and aggressive biology. However, alternative explanations for these densitometric findings must also be considered. Variations in contrast enhancement (HU) are not exclusively driven by tumor biology; they can also be significantly influenced by technical factors such as variations in cardiac output, exact timing of the venous phase acquisition, and the technical variability of manual ROI placement by the radiologist. Therefore, while the biological hypothesis of hypoxia is supported by the literature, standardized imaging protocols are strictly necessary to utilize HU density as a reliable clinical biomarker.

Our study identified that qualitative features, specifically lobulated contours and ill-defined margins, were strongly associated with high-grade histology. This radiological observation likely corresponds to the microscopic “invasive front” described by Bryne et al., where the most prognostically relevant tumor cells reside at the tumor-host interface [26]. The presence of spiculated margins on CT represents the macroscopic translation of the “spray-like” or non-cohesive pattern of invasion (POI), which is a known predictor of poor prognosis and recurrence [27,28]. This “radiological budding” suggests that simple morphological assessment can provide insight into the microscopic behavior of the tumor. Moving forward, the integration of these morphological signs with artificial intelligence may further refine risk stratification.

These findings are hypothesis-generating. Standard CECT parameters should not yet be incorporated into clinical decision-making but warrant further investigation in prospective validation cohorts. In terms of clinical feasibility, the calculation of tumor volume using the ellipsoid formula and the manual extraction of HU values are rapid processes that do not require advanced computational software like radiomics or true 3D segmentation. This makes the approach highly accessible for routine practice. While manual measurements are highly feasible and accessible in routine practice, they suffer from limited reproducibility. Manual ROI placement and qualitative assessments of margins are subjective and may not be consistently reproducible across different centers or observers. Future integration with automated, Artificial Intelligence (AI)-driven segmentation tools will likely be necessary to standardize these measurements and reduce inter-reader variability before widespread clinical adoption.

## 5. Limitations

The interpretation of these results must be considered within the context of the study’s limitations. First, the retrospective, single-center design introduces inherent selection biases. Second, our cohort was anatomically heterogeneous, including both oral cavity (76.2%) and laryngeal/maxillary (23.8%) tumors. Third, our study lacks an external validation cohort, meaning these thresholds cannot be generalized yet. Fourth, we utilized standard Hounsfield Units rather than advanced perfusion CT or texture analysis; however, this was a deliberate choice to ensure the findings remain applicable to centers with standard, universally available CT protocols. Fifth, the relatively small sample size (*n* = 42) limits the immediate generalizability of these findings. This restricted cohort size was a direct consequence of our highly stringent inclusion criteria, which required perfectly standardized preoperative CECT protocols and the absence of dental artifacts. While this limited the number of participants, it ensured a standardized technical acquisition for the imaging dataset, minimizing confounding technical variables. Therefore, this study serves as a robust proof-of-concept, and future prospective, multi-center studies with larger cohorts are warranted to validate these exact volumetric thresholds across diverse clinical settings. The limited number of outcome events (9 cases of pN+ metastasis) precluded the use of multivariate regression analysis. Therefore, we were unable to control for potential clinical confounders, and the reported univariate associations should be interpreted with caution. Because this was an exploratory pilot study enforcing highly stringent inclusion criteria (requiring standardized bi-phasic CECT, exclusion of artifacts, and complete surgical neck staging), an a priori sample size calculation was not performed. Regarding radiological assessments, we utilized the ellipsoid formula for volumetric estimation rather than true 3D slice-by-slice tumor segmentation. While the ellipsoid method was deliberately chosen for its speed and practicality in routine workflows, it may be less precise for highly irregularly shaped tumors. Finally, while two senior radiologists evaluated the scans, we did not calculate formal interobserver variability metrics (e.g., Cohen’s Kappa), and manual Region of Interest (ROI) placement remains subject to potential technical variability.

## 6. Conclusions

This exploratory study suggests that preoperative standard CECT parameters—specifically 3D tumor volume and contrast wash-in dynamics—may act as potential, non-invasive imaging markers of HNSCC aggressiveness. Notably, we identified preliminary, hypothesis-generating volumetric thresholds: a volume ≥ 6.76 cm^3^ is a highly sensitive predictor for regional lymph node metastasis (pN+), while a volume ≥ 9.43 cm^3^ is indicative of high-grade (G2/G3) histology. Furthermore, our findings highlight a paradoxical inverse relationship where more aggressive lesions exhibit reduced contrast enhancement, reflecting disorganized neo-angiogenesis and internal necrosis. These quantitative metrics, combined with qualitative morphological markers (ill-defined and lobulated margins), provide a complementary radiological tool for the entire tumor. Future prospective, multi-center studies are warranted to validate these specific thresholds and to explore the integration of these standard imaging metrics with artificial intelligence and molecular biomarkers. Ultimately, translating these non-invasive tools into clinical workflows may complement histopathological assessment and support future risk stratification in HNSCC, following rigorous prospective validation.

## Figures and Tables

**Table 1:** Baseline Demographic, Clinical, and Radiographic Characteristics of the Study Population.

Characteristic	*n* = 42
**Demographics**	
Age (years), Median [IQR]	61 (54–67)
Sex, Male, *n* (%)	35 (83.3)
**Clinical Characteristics**	
Anatomical Site, *n* (%)	
Oral Cavity	32 (76.2)
Larynx/Hypopharynx	8 (19)
Maxilla/Sinus	2 (4.8)
T-Stage (Pathological), *n* (%)	
T1/T2 (Early)	12 (54.5)
T3/T4 (Advanced)	10 (45.5)
Histological Grade, *n* (%)	
G1 (Well-differentiated)	5 (11.9)
G2 (Moderately differentiated)	36 (85.7)
G3 (Poorly differentiated)	1 (2.4)
**CT Morphological Features**	
Tumor Volume (cm^3^), Median [IQR]	16.1 (6.2–38.5)
Margin Type, Ill-defined, *n* (%)	33 (78.6)
Spiculated Borders, *n* (%)	19 (45.2)
Lobulations, *n* (%)	34 (81)
Necrosis, Present, *n* (%)	25 (59.5)
**CT Density Values (HU)**	
Native Density, Median [IQR]	35.5 (29–39)
Contrast Density, Median [IQR]	57.5 (48–65)
HU Delta (Wash-in), Median [IQR]	23 (19–27)

IQR = Interquartile Range (25th–75th percentile); HU = Hounsfield Units; *n* = Number of patients.

**Table 2:** Association between Preoperative CT Features and Histological Grade.

CT Characteristic	Low Grade (G1) (*n* = 5)	High Grade (G2/G3) (*n* = 37)	*p*-Value
Quantitative Metrics.			
Tumor Volume (cm^3^), Median [IQR]	2.9 (1.9–8)	18.1 (8.2–43.6)	0.006
Native Density (HU), Median [IQR]	39 (35–39)	34 (28–38)	0.269
Contrast Density (HU), Median [IQR]	68 (67–69)	55 (48–62)	0.010
HU Delta (Wash-in), Median [IQR]	30 (28–36)	23 (19–25)	0.008
Margin Score, Median [IQR]	0 (0–1)	1 (1–2)	0.004
Necrosis Score, Median [IQR]	0 (0–0)	1 (0–3)	0.049
Qualitative Features, *n* (%)			
Ill-defined Margins	2 (40)	31 (83.8)	0.051
Lobulated Contour	2 (40)	32 (86.5)	0.040
Spiculated Borders	0 (0)	19 (51.4)	0.053
Necrosis	1 (20)	24 (64.9)	0.140

Data presented as Median (Interquartile Range) for continuous variables and *n* (%) for categorical variables. HU = Hounsfield Units. *p*-values calculated using Exact Mann–Whitney U test for continuous variables and Fisher’s Exact test for categorical variables.

**Table 3:** Correlation between Preoperative CT Features and Lymph Node Metastasis (pN).

CT Characteristic	Node Negative (pN0) (*n* = 13)	Node Positive (pN+) (*n* = 9)	*p*-Value
Quantitative Metrics			
Tumor Volume (cm^3^), Median [IQR]	6.2 (3.9–18.1)	25.4 (9.1–43.6)	0.036
Native Density (HU), Median [IQR]	38 (28–39)	37 (31–38)	0.896
Contrast Density (HU), Median [IQR]	62 (49–65)	58 (49–61)	0.896
HU Delta (Wash-in), Median [IQR]	23 (22–24)	21 (20–28)	0.601
Margin Score, Median [IQR]	1 (1–2)	2 (1–2)	0.073
Necrosis Score, Median [IQR]	0 (0–1)	1 (0–1)	0.376
Qualitative Features, *n* (%)			
Ill-defined Margins	9 (69.2)	8 (88.9)	0.36
Lobulated Contour	9 (69.2)	9 (100)	0.115
Spiculated Borders	5 (38.5)	6 (66.7)	0.387
Necrosis	6 (46.2)	6 (66.7)	0.415

Data presented as Median (Interquartile Range) for continuous variables and *n* (%) for categorical variables. HU = Hounsfield Units; pN = Pathological Nodal Stage. *p*-values calculated using Mann–Whitney U test for continuous variables and Fisher’s Exact test for categorical variables.

**Table 4:** Spearman’s Rank Correlation Matrix of Quantitative and Morphological CT Features.

Variable	1. Tumor Volume	2. Native HU	3. Contrast HU	4. HU Delta	5. Margin Score	6. Necrosis Score
1. Tumor Volume (cm^3^)	—					
2. Native HU Mean	−0.508 **	—				
3. Contrast HU Mean	−0.554 **	0.833 **	—			
4. HU Delta (Wash-in)	−0.441 **	0.482 **	0.862 **	—		
5. Margin Score	0.594 **	−0.682 **	−0.711 **	−0.494 **	—	
6. Necrosis Score	0.626 **	−0.808 **	−0.812 **	−0.583 **	0.724 **	—

Data represents Spearman’s rho (r) correlation coefficients. HU = Hounsfield Units. ** *p* < 0.01 (2-tailed).

## Data Availability

The data supporting the findings of this study are available from the corresponding author upon reasonable request.
